# Supplementary material for: Cuticular Hydrocarbon Plasticity in Three Rice Planthopper Species
Source: Int J Mol Sci. 2021 Jul 20;22(14):7733. doi: 10.3390/ijms22147733 (PMC8304831; doi:10.3390/ijms22147733)
Supplement: Supplementary file 1 [file ijms-22-07733-s001.zip › Supplementary information.pdf]

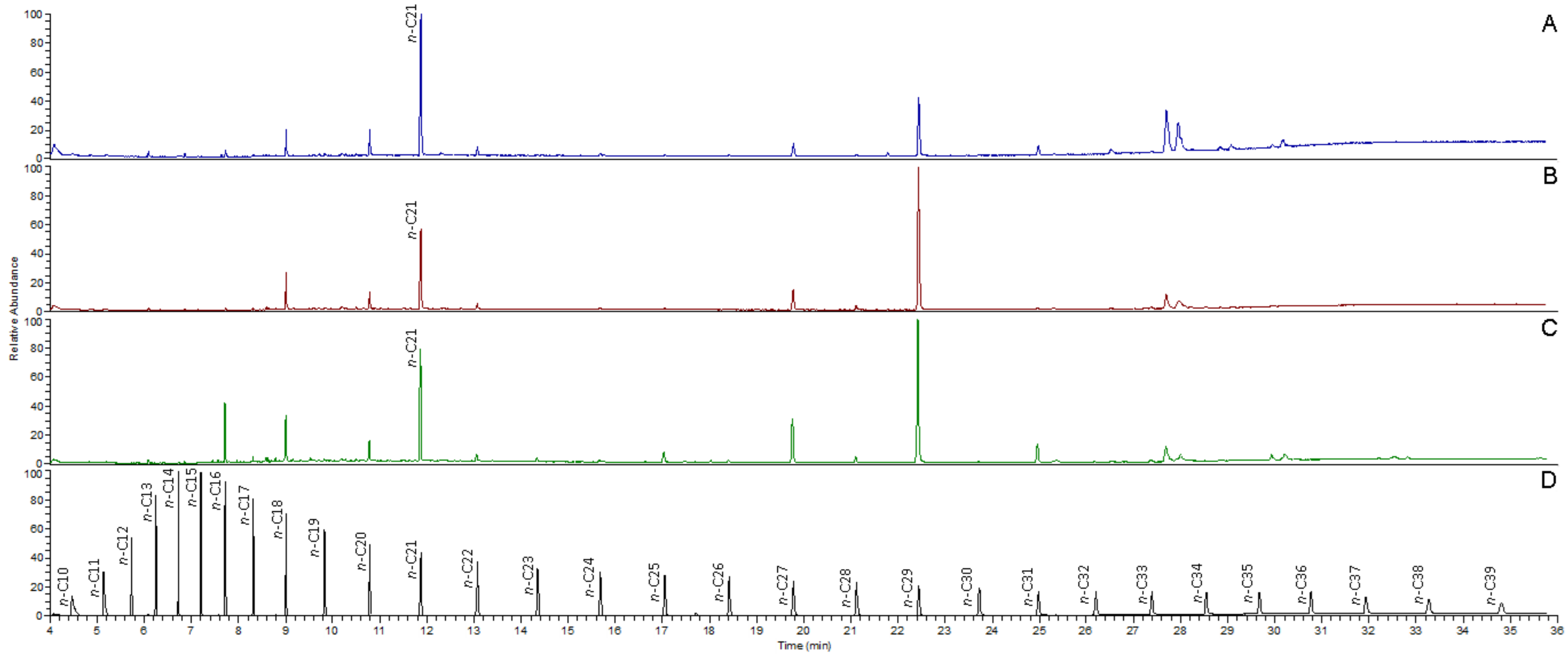

Figure S1. GC-MS analysis of CHCs. The CHC profiles are shown for the planthopper female adults (A. BPH, B. SBPH, C. WBPH, D. C_{10}-C_{39} *n*-alkanes standard).

Table S1. Composition of cuticular hydrocarbons in different developmental stages/sexes for BPH

Compound	Female adult	Male adult	5th-instar nymph	2nd- instar nymph
[a]**SumCHCs**	13.79±6.72	14.51±5.26	20.10±5.34	8.27±2.30
C_{10}	0.86 %	0.97 %	0.66 %	0.56 %
C_{11}	1.16 %	0.88 %	1.06 %	0.86 %
C_{12}	0.10 %	0.04 %	0.06 %	0.20 %
C_{13}	0.03 %	0.01 %	0.02 %	0.03 %
C_{14}	1.29 %	4.09 %	2.56 %	3.71 %
C_{15}	0.13 %	0.29 %	0.15 %	0.21 %
C_{16}	9.42 %	22.33 %	12.26 %	16.86 %
C_{17}	1.77 %	2.63 %	1.75 %	1.88 %
C_{18}	18.67 %	23.07 %	16.36 %	13.54 %
C_{19}	1.36 %	1.32 %	1.11 %	0.77 %
C_{20}	12.25 %	13.21 %	10.93 %	6.60 %
C_{22}	4.79 %	4.83 %	4.09 %	2.44 %
C_{23}	0.54 %	0.48 %	0.33 %	0.25 %
C_{24}	1.65 %	1.72 %	1.39 %	0.84 %
C_{25}	0.86 %	0.88 %	0.48 %	0.31 %
C_{26}	1.17 %	1.32 %	0.81 %	0.53 %
C_{27}	6.94 %	3.46 %	3.53 %	7.91 %
C_{28}	1.25 %	1.40 %	1.10 %	1.08 %
C_{29}	29.49 %	11.74 %	39.43 %	39.99 %
C_{30}	0.61 %	0.91 %	0.49 %	0.30 %
C_{31}	4.45 %	2.92 %	1.07 %	0.90 %
C_{32}	0.19 %	0.48 %	0.10 %	0.06 %
C_{33}	0.86 %	0.63 %	0.16 %	0.09 %
C_{34}	0.06 %	0.18 %	0.02 %	0.03 %
C_{35}	0.02 %	0.10 %	0.02 %	0.01 %
C_{36}	0.02 %	0.03 %	0.01 %	0.01 %
C_{37}	0.01 %	0.03 %	0.03 %	0.01 %
C_{38}	0.04 %	0.03 %	0.02 %	0.01 %

[a] The results were calculated from ten biological replicates (nanogram per insect ± SE).

Table S2. Composition of cuticular hydrocarbons in different developmental stages/sexes feeding on different hosts for SBPH

Compound	Female adult		Male adult		5th-instar nymph		2nd- instar nymph	
	Rice	Wheat	Rice	Wheat	Rice	Wheat	Rice	Wheat
**SumCHCs**	48.68±6.62	30.20±3.23	36.48±2.49	29.90±2.05	32.09±4.78	23.96±3.23	7.04±1.53	9.82±0.63
**C_{10}**	0.40 %	0.46 %	0.39 %	0.32 %	0.46 %	0.66 %	0.67 %	0.02 %
**C_{11}**	0.77 %	2.59 %	0.66 %	3.35 %	1.33 %	1.36 %	1.22 %	0.68 %
**C_{12}**	0.02 %	0.05 %	0.02 %	0.08 %	0.03 %	0.06 %	0.03 %	0.16 %
**C_{13}**	0.01 %	0.02 %	0.00 %	0.03 %	0.01 %	0.01 %	0.01 %	0.18 %
**C_{14}**	0.06 %	0.10 %	0.10 %	0.13 %	1.20 %	0.30 %	0.27 %	0.32 %
**C_{15}**	0.05 %	0.11 %	0.04 %	0.15 %	0.19 %	0.15 %	0.10 %	0.30 %
**C_{16}**	2.61 %	4.41 %	4.02 %	3.35 %	11.60 %	11.40 %	6.79 %	4.68 %
**C_{17}**	0.63 %	0.96 %	0.69 %	0.86 %	1.68 %	1.77 %	1.04 %	1.50 %
**C_{18}**	7.96 %	10.41 %	7.94 %	8.59 %	19.12 %	15.61 %	12.19 %	11.02 %
**C_{19}**	0.48 %	0.83 %	0.48 %	0.69 %	0.91 %	1.19 %	0.86 %	1.10 %
**C_{20}**	5.25 %	8.39 %	5.54 %	6.59 %	9.31 %	10.31 %	9.18 %	8.04 %
**C_{22}**	2.08 %	3.25 %	2.24 %	2.61 %	3.32 %	4.07 %	3.41 %	5.74 %
**C_{23}**	0.42 %	1.31 %	0.31 %	0.79 %	0.35 %	1.00 %	0.34 %	3.78 %
**C_{24}**	0.79 %	1.28 %	0.82 %	1.12 %	1.16 %	1.95 %	1.16 %	3.14 %
**C_{25}**	1.07 %	2.52 %	5.56 %	13.81 %	0.69 %	1.58 %	0.71 %	2.06 %
**C_{26}**	0.66 %	0.87 %	0.78 %	1.07 %	0.85 %	1.39 %	1.06 %	1.97 %
**C_{27}**	10.11 %	5.14 %	12.29 %	9.63 %	9.24 %	5.31 %	24.71 %	14.12 %
**C_{28}**	1.89 %	1.77 %	1.77 %	1.76 %	1.47 %	1.82 %	1.65 %	2.55 %
**C_{29}**	62.26 %	50.39 %	53.87 %	40.79 %	35.67 %	37.73 %	33.49 %	36.43 %
**C_{30}**	0.30 %	0.51 %	0.33 %	0.45 %	0.33 %	0.59 %	0.29 %	0.65 %
**C_{31}**	0.99 %	2.78 %	0.97 %	1.81 %	0.40 %	0.75 %	0.34 %	0.68 %
**C_{32}**	0.17 %	0.27 %	0.19 %	0.29 %	0.16 %	0.24 %	0.14 %	0.24 %
**C_{33}**	0.59 %	1.13 %	0.69 %	1.42 %	0.29 %	0.48 %	0.24 %	0.41 %
**C_{34}**	0.41 %	0.34 %	0.23 %	0.25 %	0.16 %	0.18 %	0.03 %	0.20 %
**C_{35}**	0.01 %	0.02 %	0.01 %	0.01 %	0.01 %	0.01 %	0.01 %	-
**C_{36}**	0.02 %	0.05 %	0.03 %	0.05 %	0.02 %	0.04 %	0.02 %	0.03 %
**C_{37}**	0.01 %	0.01 %	0.01 %	0.01 %	0.01 %	0.01 %	0.01 %	0.01 %
**C_{38}**	0.01 %	0.02 %	0.01 %	0.02 %	0.01 %	0.01 %	0.01 %	0.01 %

Table S3. Composition of cuticular hydrocarbons in different developmental stages/sexes feeding on different hosts for WBPH

Compound	Female adult		Male adult		5th-instar nymph		2nd- instar nymph	
	Rice	Wheat	Rice	Wheat	Rice	Wheat	Rice	Wheat
**SumCHCs**	51.43±2.70	60.21±7.46	47.31±6.53	48.57±5.60	42.17±5.76	38.56±2.92	9.71±1.86	16.67±3.03
C_{10}	0.24 %	0.01 %	0.27 %	-	0.33 %	0.01 %	0.35 %	0.01 %
C_{11}	0.99 %	0.35 %	2.55 %	0.50 %	3.94 %	0.70 %	4.26 %	0.12 %
C_{12}	0.02 %	0.03 %	0.04 %	0.03 %	0.07 %	0.03 %	0.07 %	0.01 %
C_{13}	-	0.04 %	0.01 %	0.03 %	0.02 %	0.06 %	0.02 %	0.02 %
C_{14}	0.14 %	0.02 %	0.04 %	0.03 %	0.11 %	0.02 %	0.06 %	0.02 %
C_{15}	0.07 %	0.03 %	0.07 %	0.04 %	0.08 %	0.05 %	0.08 %	0.03 %
C_{16}	5.32 %	0.81 %	2.48 %	1.11 %	2.31 %	0.90 %	4.93 %	0.88 %
C_{17}	0.79 %	0.49 %	0.55 %	0.50 %	0.52 %	0.65 %	0.87 %	0.47 %
C_{18}	10.25 %	5.99 %	7.79 %	5.66 %	7.21 %	8.14 %	12.26 %	4.99 %
C_{19}	0.63 %	0.70 %	0.47 %	0.62 %	0.54 %	1.05 %	0.72 %	0.58 %
C_{20}	5.74 %	4.99 %	4.78 %	4.72 %	5.96 %	7.78 %	7.32 %	4.31 %
C_{22}	2.24 %	3.12 %	1.94 %	2.99 %	2.56 %	4.86 %	2.81 %	2.69 %
C_{23}	1.74 %	2.29 %	2.47 %	2.53 %	1.44 %	3.32 %	1.68 %	2.33 %
C_{24}	0.90 %	1.43 %	0.78 %	1.43 %	1.08 %	2.16 %	1.26 %	1.27 %
C_{25}	2.79 %	2.16 %	2.58 %	2.29 %	2.72 %	2.52 %	3.95 %	3.05 %
C_{26}	0.99 %	1.08 %	1.40 %	1.45 %	1.31 %	1.26 %	1.33 %	1.09 %
C_{27}	13.86 %	13.25 %	24.63 %	21.28 %	16.92 %	12.17 %	17.29 %	16.69 %
C_{28}	2.44 %	2.97 %	2.83 %	2.92 %	2.79 %	2.86 %	2.42 %	3.03 %
C_{29}	45.72 %	53.38 %	40.46 %	45.68 %	48.11 %	48.05 %	36.81 %	55.73 %
C_{30}	0.58 %	0.72 %	0.48 %	0.76 %	0.41 %	0.81 %	0.47 %	0.52 %
C_{31}	3.64 %	4.40 %	2.46 %	3.42 %	1.09 %	1.62 %	0.71 %	1.29 %
C_{32}	0.21 %	0.41 %	0.18 %	0.42 %	0.14 %	0.30 %	0.12 %	0.23 %
C_{33}	0.57 %	1.19 %	0.70 %	1.50 %	0.29 %	0.59 %	0.18 %	0.54 %
C_{34}	0.05 %	0.05 %	0.01 %	0.03 %	0.01 %	0.04 %	-	0.02 %
C_{35}	0.01 %	0.04 %	0.01 %	0.04 %	0.01 %	0.02 %	-	0.02 %
C_{36}	0.05 %	0.02 %	0.01 %	0.03 %	0.01 %	0.01 %	0.01 %	0.01 %
C_{37}	0.01 %	0.01 %	0.01 %	0.01 %	0.01 %	0.01 %	-	0.01 %
C_{38}	0.01 %	0.02 %	-	0.01 %	0.01 %	0.02 %	0.01 %	0.01 %
